# Eye movements in patients with Whiplash Associated Disorders: a systematic review

**DOI:** 10.1186/s12891-016-1284-4

**Published:** 2016-10-21

**Authors:** Britta Kristina Ischebeck, Jurryt de Vries, Jos N Van der Geest, Malou Janssen, Jan Paul Van Wingerden, Gert Jan Kleinrensink, Maarten A Frens

**Affiliations:** 1 Spine and Joint Centre, Noordsingel 113, 3035 EM Rotterdam, The Netherlands; 2 Department of Neuroscience, Erasmus MC, Rotterdam, The Netherlands; 3 Department of Manual Therapy, Rotterdam University of Applied Sciences, Rotterdam, The Netherlands; 4 Erasmus University College, Rotterdam, The Netherlands

**Keywords:** Whiplash Associated Disorders (WAD), Problems with vision, Oculomotor problems, Systematic review

## Abstract

**Background:**

Many people with Whiplash Associated Disorders (WAD) report problems with vision, some of which may be due to impaired eye movements. Better understanding of such impaired eye movements could improve diagnostics and treatment strategies.

This systematic review surveys the current evidence on changes in eye movements of patients with WAD and explains how the oculomotor system is tested.

**Methods:**

Nine electronic data bases were searched for relevant articles from inception until September 2015. All studies which investigated eye movements in patients with WAD and included a healthy control group were screened for inclusion. Qualifying studies were retrieved and independently assessed for methodological quality using the Methodology Checklists provided by the Scottish Intercollegiate Guidelines Network.

**Results:**

Fourteen studies out of 833 unique hits were included. Ten studies reported impaired eye movements in patients with WAD and in four studies no differences compared to healthy controls were found. Different methods of eye movement examination were used in the ten studies: in five studies, the smooth pursuit neck torsion test was positive, in two more the velocity and stability of head movements during eye-coordination tasks were decreased, and in another three studies the cervico-ocular reflex was elevated.

**Conclusions:**

Overall the reviewed studies show deficits in eye movement in patients with WAD, but studies and results are varied. When comparing the results of the 14 relevant publications, one should realise that there are significant differences in test set-up and patient population. In the majority of studies patients show altered compensatory eye movements and smooth pursuit movements which may impair the coordination of head and eyes.

## Background

People who suffer from chronic ‘Whiplash Associated Disorders’ (WAD) exhibit very distinct complaints [1]. Seventy percent of patients complain of pain, dizziness and unsteadiness [2], while 50 % report problems with vision [3]. These problems with vision comprise concentration problems during reading, sensitivity to light, visual fatigue and eye strain [3]. The severity of problems with vision is higher in traumatic neck pain patients than in non-traumatic neck pain patients [3]. Problems in vision could be due to malfunction of the oculomotor system that is meant to keep the eye on a target [4, 5]. Such oculomotor problems in WAD patients could be related to cervical sensorimotor disorders. The knowledge of cervical induced oculomotor system disorders is still limited [6]. This may be because of the complexity of the cervico-oculomotor system, that includes not only the central nervous system but also the proprioceptive system of the cervical spine (for review see e.g. [7]).

Eye movement control depends on eye position in the head and on the position of the head in space [8]. Head position is determined by integration of several sub-systems such as the vestibular system, visual information and proprioceptive system of the cervical spine [8, 9]. Disturbed afferent cervical information is related to nystagmus, dizziness and deficits in balance [10, 11].

The principal source of cervical afferent information is formed by mechanoreceptors in the upper cervical spine. Specifically in the deep upper cervical muscles (i.e. m. obliquus capitis superior and inferior, m. longus colli), the density of muscle spindles is extremely high compared to other muscles in the body [12, 13]. Muscle spindles are part of the sensorimotor system [14]. In patients with WAD sensorimotor control is disturbed [14–17].

In attempts to reveal the complex relation between cervical sensorimotor disorders and visual problems several studies regarding oculomotor problems in patients with WAD have been published [3, 18–23]. In all studies one of three distinct eye movement types were used to assess oculomotor problems in patients with WAD: eye stabilization reflexes, smooth pursuit eye movements and head-eye coordination.

### Eye stabilisation reflexes

Eye stabilization reflexes preserve stable vision on the retina during head movement. At least three eye stabilization reflexes can be distinguished based on their sensory input: the cervico-ocular reflex (COR), the vestibulo-ocular reflex (VOR) and the optokinetic reflex (OKR). These three complementary reflexes have distinct characteristics and receive input from the cervical spine, the vestibulum and the eyes, respectively. The COR receives input from muscle spindles in the cervical spine, especially from the deep upper cervical muscles and joint capsules of C1 to C3 [24]. The central pathways of the VOR and the COR are the same; both reflexes converge at the vestibular nuclei [24]. The OKR pathways, however, are quite distinct from the COR and VOR pathways [25].

### Smooth pursuit eye movements

Accurate smooth pursuit is essential to look at a moving object by keeping the retinal image steady within the foveal area. Ideally, smooth pursuit velocity matches the velocity of the moving object. Performing smooth pursuit eye movements properly requires the integration of visual, vestibular and cervical information [26].

### Head-eye coordination

Head-eye coordination is the overall result of all systems in control of the visual system. During these tasks, the compensatory eye movements and the motor control of the neck co-operate, requiring integration of saccades, the COR, VOR, OKR and active neck movements.

This systematic review provides an overview of existing evidence on oculomotor system changes in patients with WAD and how this evidence was perceived. We aim to address the question of what is known about changed eye movements in patients with WAD. To our knowledge no reviews of the literature concerning oculomotor problems in patients with WAD have previously been published. Therefore, we present a comprehensive, systematic overview of the literature concerning changed eye movements in patients with WAD compared to healthy controls.

## Methods

The PRISMA guidelines (Preferred Reporting Items for Systematic Reviews and Meta-Analyses) were employed in this systematic literature review [27].

### Information sources and search parameters

To be as comprehensive as possible, the following databases have been searched until September 2015: Embase, Medline (OvidSP), Web of Science, Scopus, Cinahl, SportDiscus, Cochrane, Pubmed Publisher and Google scholar. Keywords were derived from the research question and transformed to associated and free text words. The search strategy in Embase was based on the following combination of terms: ‘cornea reflex’/exp OR ‘eye movement’/exp OR ‘eye movement disorder’/de OR ‘oculomotor system’/de OR ‘extraocular muscle’/de OR (((cornea* OR eye* OR ocular* OR cervicoocul* OR visual*) NEAR/6 (reflex* OR movement* OR pursuit* OR motilit* OR track*)) OR oculomotor* OR ((extraocular* OR ocular* OR eye*) NEAR/3 muscle*) OR ‘smooth pursuit’ OR (tracking NEAR/3 (perform* OR task*))):ab,ti) AND (‘neck pain’/de OR ‘neck injury’/de OR ‘whiplash injury’/exp OR (((neck OR cervic* OR colli OR collum*) NEAR/6 (pain* OR hyperextension* OR ache OR injur* OR disorder* OR trauma* OR lesion* OR bruise*)) OR neckache* OR Cervicalgia* OR Cervicodynia* OR whiplash):ab,ti).

In addition, Medline (OvidSP), Web of Science, Scopus, Cinahl, SportDiscus, Cochrane, Pubmed Publisher and Google scholar were similarly searched with their own thesaurus used for indexing articles and free entries.

### Study selection

For inclusion in the systematic review the following criteria had to be met: (1) participants in the study had to be 18 years or older; (2) patients had to have Whiplash Associated Disorders; (3) one of the outcome measures in the study had to be eye movements; (4) control subjects were healthy individuals; (5) the article was written in English, Dutch or German; (6) the original article was available in full text.

### Data items and collection

Information was extracted from the included articles and presented in the evidence table (Table 1), regarding (1) study, (2) sample size, (3) characteristics of the patients, (4) testing device for eye movements, (5) eye movements testing protocol, (6) results and (7) possible bias.

**Table 1:** Evidence table of the included studies

Reference	Sample	Inclusion criteria	Testing instrument	Testing protocol	Results	Possible bias
Dispenza et al., 2011 [33]	33 WAD (36.5y, 21–53) 23 CON (30.4y, 19–49)	WAD (without loss of consciousness) 1–12 months after accident	video-oculography: velocity 18°/s; fully- automated analysis	SPNT	neutral: WAD 0.86, CON 0.87 torsion to the right : WAD 0.87 torsion to the left: WAD 0.86 SPNT-diff: WAD 0	type of WAD not described, selection of controls not described, no SP in relatively rotated position tested in controls
Grip et al., 2009 [39]	6 WAD (28y) 20 CON (32y)	WAD >3 months after accident	electro-oculography	gaze stability; sequential eye and head movement (SEHM)	gaze stability: WAD: head angle reduced (no exact data) SHEM: WAD: mean angular head velocity reduced (no exact data)	small population (*n* = 7) no individual data, results presented in boxplots
Heikkila et al., 1998 [20]	27 WAD (38.8y, 18–66) 25 CON (34y, 25–40y)	acute WAD II, III (without loss of consciousness)	electro-oculography: velocity 20°/s and 30°/s; fully- automated analysis	SP	30° right rotation of the eyes: WAD 2× abnormal; 30° left rotation of the eyes: WAD 5× abnormal; 20° right rotation of the eyes: WAD 2× abnormal; 20° left rotation of the eyes: WAD 0× abnormal	only SP in neutral position tested, no torsion of the neck only quantity of abnormal scores provided, no individual data
Janssen et al., 2015 [40]	11 WAD, 44 non-WAD (44.2y, 25–67) 20 CON (28.4, 20–51)	WAD > 6 months after accident	video- oculography: frequency 0.4Hz; 0°, 15°, 30° and 45° chair position; predictable and unpredictable stimuli; semi- automated analysis	SPNT	SPNTdiff predictably: WAD 0.08, non-WAD 0.05, CON 0.02 SPNT unpredictably: WAD 0.01, non-WAD 0.01, CON 0	no specification of grade of WAD
Kelders et al., 2005 [23]	8 WAD (32y, 25–42) 8 CON (35y, 30–45)	WAD I, II, III 5–36 months after accident	video- oculography	cervico-ocular reflex	COR higher in WAD than in CON^a^	little data provided, only graphs
Kongsted et al., 2007 [36]	34 WAD (39.4y, 20–51) 60 CON (40y, 18–63))	WAD I, II, III > 6 months after accident	electro-oculography: max velocity 37°/s; frequency 0.2Hz; fully- automated analysis	SPNT	neutral: WAD 0.9, CON 0.96 (median) torsion to the right: WAD 0.89, CON 0.94 torsion to the left: WAD 0.93, CON 0.95 SPNTdiff: WAD 0, CON 0	patient population heterogeneous regarding symptoms, disability and duration of symptoms
Montfoort et al., 2006 [21]	13 WAD (40y, 26–60) 18 CON (36y, 23–64)	WAD I, II	video-oculography	cervico-ocular reflex; vestibulo-ocular reflex; optokinetic reflex	COR: *P* = 2.9 × 10^−6a^ VOR: *P* = 0.27 OKR: *P* = 0.25	only comparison between groups, no individual data
Montfoort et al., 2008 [22]	COR: 10 WAD (42y, 22–52), 10 CON (31y, 18–54) VOR: 10 WAD (39y, 19–56), COR 30y, 24–39)	WAD I, II	video-oculography	cervico-ocular reflex; vestibulo-ocular reflex; optokinetic reflex	COR adaptation: WAD ∆G = 0.13 ± 0.24, CON ∆G = −0.19 ± 0.06^a^ VOR adaptation: WAD ∆G = 0.037 ± 0.062, CON ∆G = −0.2 ± 0.072^a^	no comparison between characteristics of patients and controls, little data provided
Prushansky et al., 2004 [35]	26 WAD (40.3y, 25–55) 23 CON (34.2y, 18–54)	WAD II, III 6–84 months after accident	electro-oculography: velocity 0.75 m/s; no information about type of analysis	SPNT	neutral: WAD 0.79^a^, CON 0.86 torsion to the right: WAD 0.74, CON 0.82 torsion to the left: WAD 0.75, 0.80 SPNTdiff: WAD 0.026, CON 0.035	remarkable variation in duration of neck pain; no information about velocity in degrees per second
Tjell et al., 1998 [34]	50 WAD D (39y, 18–60) 25 WAD ND (34y, 21–63)	≥ WAD II > 6 months after accident	electro-oculography: velocity 20°/s; frequency 0.2Hz; manual analysis	SPNT	SPNTdiff: WAD D 0.14^a^; WAD ND 0.10^a^; CON 0.02	vague exclusion criteria for controls: tension in neck
Treleaven et al., 2005 [37]	100 WAD: 50 WAD D (35y, 19–46) 50 WAD ND (35y, 18–46) 50 CON (30y, 19–45)	WAD II > 3 months after accident	electro-oculography: velocity 20°/s; frequency 0.2Hz; semi- automated analysis	SPNT	neutral: WAD ND 0.82, CON 0.88^a^ torsion to the right: WAD ND 0.78, CON 0.88^a^ torsion to the left: WAD ND 0.74, CON 0.87^a^ SPNTdiff: WAD D 0.11^a^; WAD ND 0.07, CON 0.01^a^	
Treleaven et al., 2006 [16]	50 WAD D (35.5y, 19–46) 50 WAD ND (35y, 18–46) 40 CON (29.6y, 19–45)	WAD II > 3 months after accident	electro-oculography: velocity 20°/s; frequency 0.2Hz; semi- automated analysis	SPNT	WAD D: 45 abnormal SPNT scores WAD ND: 39 abnormal SPNT scores	only quantity of abnormal scores provided, no individual data
Treleaven et al., 2008 [41]	20 WAD (46.5, 40–60) 20 CON (49.5y, 43–59)	WAD with dizziness, > 3 months after accident	electro-oculography: velocity 20°/s; frequency 0.2Hz; semi- automated analysis	SPNT	SPNTdiff: WAD ~0.12^a^; CON ~ <0.02^a^	no specification of grade of WAD; only graphic presentation of data, no exact values
Treleaven et al., 2011 [19]	20 WAD (37y) 20 CON (33y)	WAD symptoms >3 months, < 5 year	electro-oculography	gaze stability; sequential eye and head movement (SEHM)	gaze stability: WAD 27.7^a^/30.5^a^, CON 44.5/43.5 (degrees of head ROM right and left) WAD 16.9/20.2, CON 33.0/37.4^a^ (head rotation velocity in degrees/s) SHEM: WAD 23.6/30, CON 36.9^a^, WAD 30, CON 36.9/36.9^a^ (head rotation velocity in degrees/sec)	remarkable variation in duration of neck pain

*WAD* Whiplash associated disorder, WAD grade I neck complaints of pain, stiffness or tenderness only but no physical signs are noted by the examining physician, WAD grade II neck complaints and musculoskeletal signs as decreased range of motion and point tenderness in the neck, WAD grade III includes additional signs (decreased or absent deep tendon reflexes, weakness, and sensory deficits), *WAD D* patients with WAD and dizziness, *WAD ND* patients with WAD without dizziness, *CON* healthy controls, *y* mean years of age, *SPNT* Smooth Pursuit Neck Torsion Test, *SP* smooth pursuit, *SPNTdiff* difference in SP gain between neutral and relatively rotated position, *COR* cervico-ocular reflex, *VOR* vestibulo-ocular reflex, *ROM* cervical range of motion, *SEHM* sequential eye and head movement, ^a^ indicates statistically significant differences between groups

### Risk of bias in individual studies

The validity and risk of bias of the included articles was checked by using the “Methodology Checklist 4: Case–control studies” version 2.0 and “Methodology Checklist 3: Cohort studies” version 3.0 provided by the Scottish Intercollegiate Guidelines Network (SIGN). The risk of bias table is presented in Table 2. The appraisal of the articles was based on the description of the internal validity, i.e. the selection of subjects, exclusion of selection bias, clear definition of outcomes, blinding of assessors, reliable assessment of exposure, identification of potential confounders and provision of confidence intervals. For the studies the grading score has been set from “Low quality” (0), “Acceptable” (+) or “High quality” (++). In the present review, only articles graded as “Acceptable” or “High quality” were included. This criterion was set a priori.

**Table 2:** Risk of bias table presenting individual criteria in SIGN checklists for the 14 included studies

	Internal validity: selection of subjects							Internal validity: assessment		Internal validity: confounding	Internal validity: statistical analysis	Overall assessment		
	Appropriate research question	Cases and controls from comparable population	Same exclusion criteria	Percentage of each group participating in the study	Comparison between participants and non-participants	Cases are clearly defined and differentiated from controls	Established that controls are non-cases	Prevention of primary exposure influencing case ascertainment	Standard, valid and reliable exposure	Identification of main potential confounders	Confidence intervals	Minimization the risk of bias	Clear association between exposure and outcome	Results directly applicable to patient group
Dispenza et al., 2011 [33]	+	?	-	Cases: 89 % Controls: 100 %	-	-	-	?	+	?	+	-	-	-
Grip et al., 2009 [39]	+	+	+	Cases: 100 % Controls: 90 %	+	+	+	d.n.a	+	?	+	++	+	+
Heikkila et al., 1998 [20]	+	?	+	Cases: 100 % Controls: 100 %	-	+	+	d.n.a.	-	+	+	+	+	+
Janssen et al., 2015 [40]	+	+	-	Cases: 99 % Controls: 100 %	-	+	+	d.n.a.	+	?	+	+	+	+
Kelders et al., 2005 [23]	+	?	?	Cases: 100 % Controls: 100 %	-	-	?	d.n.a.	+	+	+	+	+	+
Kongsted et al., 2007 [36]	+	+	+	Cases: 70 % Controls: 90 %	+	+	+	+	+	+	+	++	+	+
Montfoort et al., 2006 [21]	+	+	-	Cases: 100 % Controls: 100 %	-	?	?	?	+	?	-	+	-	-
Montfoort et al., 2008 [22]	+	?	?	Cases: 95 % Controls: 100 %	+	+	+	?	+	?	+	+	+	+
Prushansky et al., 2004 [35]	+	+	+	Cases : 100 % Controls : 100 %	-	+	+	d.n.a.	+	-	+	+	+	+
Tjell et al., 1998 [34]	-	+	-	Cases: 75 % Controls: 100 %	+	?	+	d.n.a.	+	+	+	+	+	-
Treleaven et al., 2005 [37]	+	?	?	Cases: 100 % Controls: 100 %	-	+	+	+	+	+	+	+	-	-
Treleaven et al., 2006 [16]	+	+	+	Cases: 100 % Controls: 100 %	+	+	+	+	+	?	-	+	+	+
Treleaven et al., 2008 [41]	+	+	-	Cases: 100 % Controls: 100 %	+	+	+	?	?	+	-	+	+	+
Treleaven et al., 2011 [19]	+	?	+	Cases: 100 % Controls: 100 %	+	+	+	?	+	+	+	++	+	+

+ = yes; - = no; ++ = high quality; + = acceptable; - = unacceptable; d.n.a. = does not apply

Methodological quality of the included articles was assessed blindly and independently by authors BI and JV. After both researchers appraised the selected articles, results were compared and any differences discussed after screening the article a second time.

## Results

### Study selection

**Fig. 1:**
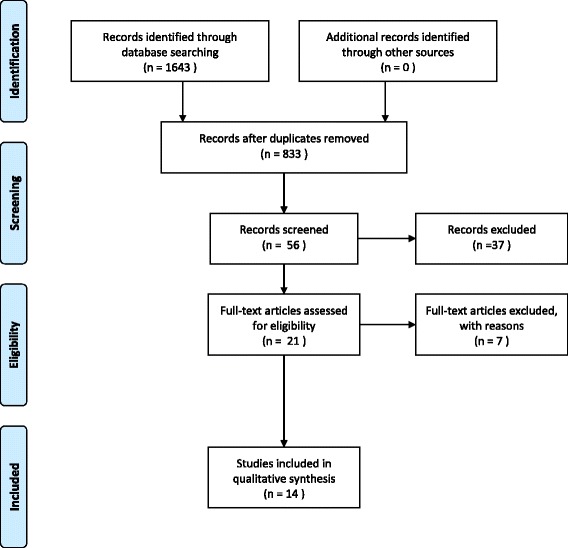
PRISMA flow diagram

A total of 833 studies were identified. As shown in Fig. 1, 14 studies remained after two screening phases.

In the first phase all articles were screened on relevance of the title and abstract. Twenty-one of the included studies remained after the first screening. These studies met the inclusion criteria, according to the title and abstract. After the first full-text reading, two researchers agreed on 19 of the 21 studies. Seven of these 19 studies were excluded because they did not fulfil the inclusion criteria, regarding the participants [28–30] or the outcome parameter [3, 18, 31, 32].

In two studies, the reviewers disagreed on the validity of the measurement protocol [33, 34]. After a second reading and comparison of the differences, the researchers reached consensus. Both studies were included, resulting in 14 included studies.

The methodological quality of all of the included studies was “acceptable” (+) according to the SIGN criteria checklist. This implies some weaknesses in the study, with an associated risk of bias. Most studies used rather small and heterogeneous populations (e.g. the time after accident of the patients varied from 1 month to 7 years [33, 35]). There was also limited information concerning raw data, possible confounders and patient characteristics (e.g. pain, anxiety and disability).

### Study characteristics

The characteristics of the data that were extracted from the included studies (study, sample size, characteristics of the patients, eye movement testing instrument, testing protocol, results, and possible bias) are presented in Table 1.

Thirteen studies were case control studies and one was a cohort study [20].

Nine studies used the classification of the Quebec Task Force on Whiplash Associated Disorders (WAD) [16, 20–23, 34–37]. In these studies patients were included with WAD grade 1 (complaints of neck pain, stiffness or tenderness only without physical signs that are noted by an examining physician), grade 2 (complaints of neck pain and musculoskeletal signs, such as a decreased range of motion and point tenderness in the neck) or grade 3 (includes additional signs such as decreased or absent deep tendon reflexes, weakness, and sensory deficits) [38].

All 14 studies included a healthy control group.

### Outcome measures

The principal outcome measure of the current review was eye movements, being the main subject of investigation in all included studies. However, different tests for eye movements were used among the included studies. The different tests were: (1) tests for head-eye coordination, integrating compensatory eye movements and neck movement tests; (2) smooth pursuit tests and (3) compensatory eye movement tests, including the VOR and the COR. Also for these three different tests, two different eye movement measurement techniques were used: electro-oculography and video-oculography.

#### Head-eye coordination

In two studies several parameters concerning the head-eye coordination were tested using two different tests [19, 39]. One of the tests was gaze stability during active head rotation. The other test was the sequential head and eye movement (SHEM) test. During the gaze stability test, the subject has to keep the eyes focussed on a point straight ahead while rotating the neck actively. During the SHEM test, the subject has to move the eyes first to one side, followed by an active head motion. Subsequently the subject first moves the eyes and then the head back to the starting position. During these tasks the compensatory eye movements and the motor control of the neck co-operate, requiring integration of saccades, the COR, VOR, OKR and active neck movements. In both tasks the patients executed the head movements slower compared to controls. During the gaze stability test, head range of motion was smaller in patients.

#### Smooth pursuit eye movements

Nine studies used smooth pursuit eye movements with a large variety in patient selection and study set-up (for details see Table 1) [16, 20, 33–37, 40, 41]. This variety complicates proper comparison of the studies. In addition, in some studies set-up information is incomplete. The evidence table (Table 1) shows possible bias of each selected study.

One study tested the smooth pursuit eye movements in neutral position only and not in a torsioned neck position. However they used two different velocities [20]. When tested with 20°/s only two, and with 30°/s five of the 26 patients were classified with dysfunctional gain (i.e. the ratio between the movement of the eyes and the movement of the stimulus).

In all other studies, using varying velocities between 18°/s and 37°/s, in contrast to the first study, the influence of a relative rotated cervical spine (head stationary, trunk turned) on the smooth pursuit eye movement was tested (smooth pursuit neck torsion (SPNT) test) [16, 33–37, 40, 41].

In four of the eight studies the primary outcome parameter (‘SPNTdiff’ the difference between the gain in neutral and in relatively rotated position) was significantly higher in patients compared to healthy controls (WAD 0.14/0.11/0.08/0.12, controls 0.02/0.01/0.02/0.02) [34, 37, 40, 41]. All four mentioned studies manually analysed the data and excluded all blinks and square waves [34, 37, 41] and saccades [40]. Three other studies did not find any differences between cases and controls [33, 35, 36]. In the later three studies the data was analysed fully-automated [33, 36] and one study does not provide information about the analysis [34].

Another study provided only the number of patients with an altered SPNTdiff compared to controls, but did not provide the median values of the smooth pursuit gain [16]. In one study with a semi-automated analysis the SPNT difference of patients with WAD was larger for predictably moving targets compared to unpredictably moving targets. This difference was not seen in healthy controls and patients with non-traumatic neck pain [40].

#### Eye stabilization reflexes

In three studies the COR and the VOR were measured [21–23]. These eye stabilization reflexes were tested in a custom setting with an infrared eye tracking device in a darkened room (further description of the measurement method in [42]). All studies reported a significantly higher COR gain in patients with WAD. One study described that both the COR and VOR gain could adapt in healthy controls, but not in patients [22].

In summary, as shown in Table 1, ten of the fourteen studies reported differences between patients with WAD and healthy controls [16, 19, 21–23, 34, 37, 39–41]. Velocity of eye movements is decreased and eye movements are less coordinated in patients than in healthy controls. In five of the eight studies which used the SPNT test, the smooth pursuit movements in the neck-rotated position were slower in the patient group compared to the healthy controls [16, 34, 37, 40, 41]. In all five studies which used the tests for eye stabilization reflexes and the head-eye coordination tests, the WAD group performed worse than the healthy control group [19, 21–23, 39]. In the discussion section we will discuss extensively the variety of outcome parameters in the tests for oculomotor deficits. Generally, patients with WAD had an elevated COR and had more problems in stabilizing the head and gaze during stability tasks and sequential movement tasks.

The differences and possible shortcomings of all studies are summarized in Tables 1 and 2. Four studies did not find differences between patients and healthy controls [20, 33, 35, 36]. All four studies analyzed smooth pursuit movements. In these studies the way of data analysis varied (two times fully automated, one time semi-automated and one time not specified), which was mentioned in one study as possible reason [36]. It is also noteworthy that the studied population was very heterogeneous or insufficiently described. Two studies mentioned this differences in symptom severity of the patient group and also attentional deficits of the patients as possible reasons [33, 35]. Heikkilä et al. found differences in patients after a whole battery of oculomotor tests, but no differences in the smooth pursuit test alone [20]. In general, most studied studies lack details in the description of patient characteristics [16, 20–23, 33–35, 39–41]. Heterogeneity in patient population may be an important factor in confounding the results of eye movement tests.

## Discussion

The current review provides an overview of present knowledge on altered eye movements in WAD patients. The majority of studies in this review confirm the possibility of eye movement impairments in WAD patients. This underlines the necessity to include an examination of eye movement impairments in the diagnostic process of patients with WAD. There are various methods that address different aspects of eye movement. The 14 studies included in this review are evaluated by the specific aspect of oculomotor problems that are tested, their clinical applicability and test validity.

### Head-eye coordination

Two studies used a series of tests to analyse the head-eye coordination [19, 39]. The purpose of this method is to evaluate over-all head-eye coordination disturbances. This method does not allow discrimination as to which part of the system is causing the actual disturbance. The head-eye coordination tests were developed for clinical use, are well described and relatively easy to execute. However, due to the requirement of active cervical movements and the combination of cervical, vestibular and visual input, it is not possible to draw specific conclusions about eye movements in isolation. The studies included in this review did not provide substantial information on the validity of this method. However, in another study that was excluded from this review as it was not performed on WAD patients the discriminative validity and reliability were considered sufficient when three out of five test scored positive [43].

### Smooth pursuit eye movements

Eight studies focused on smooth pursuit eye movements by using the SPNT test [16, 33–37, 40, 41]. The SPNT test is developed for clinical use and eye movements are measured with electro-oculography. One point of concern is the diversity in analysing the recordings. The accuracy, reliability and non-standardized interpretation is a source of bias [36, 44, 45]. In this review the four studies that did not find differences between patients with WAD and healthy subject were all SPNT test studies. This leads to the conclusion that the used analysis of the SPNT test is possibly not optimal and has to be developed. Until then the SPNT test should be used with care in the clinical setting.

In addition, as in the head-eye coordination method, it remains unclear what exactly is causing the recorded disturbance. In a recent study on the SPNT test the question was raised whether confounding factors such as pain experience or impaired cognitive functioning may affect test outcomes [40]. However, Treleaven et al. found no association to SPNT with pain, anxiety, medication, level of disability and time since injury [37]. More research seems to be required into the effect of patient characteristics on eye movements.

### Eye stabilization reflexes

Solitary cervical induced eye movements were investigated in three studies. These studies focused on eye stabilization reflexes and measured the COR in isolation. COR gain was measured without influence of visual, vestibular or cervical motor information [21–23]. Therefore it is impossible to influence COR gain deliberately, which makes the COR an objective outcome measure of oculomotor function. However, the experimental setup for the COR test is complex and it is necessary to perform the test in a completely darkened room.

A future challenge would be the conversion of the existing test into a less expensive and easy to perform test, suitable for the clinical practice. Recording of eye stabilization reflexes is relatively new. The present studies provide little information on validity of the test.

In general, comparing all three methods in one patient group may clarify which methods are most applicable to evaluate oculomotor problems in patients with WAD. At present head-eye coordination measurements seem the most suitable for clinical use. Particularly when training head-eye disturbances is used as therapeutic intervention. When a test comprises multiple (sub-) systems, it remains difficult to determine the most important factor in the observed change. However, this knowledge is necessary for successful treatment of the patient. To enhance therapeutic interventions, more insight in aetiological relations between WAD and oculomotor dysfunction is essential. At present, eye stabilization reflexes, more than the smooth pursuit method, may enhance our comprehension of the complex interaction between the cervico-oculomotor system and the coherence of neck pain symptoms. The used methodology of the SPNT test varies widely. Before using the smooth pursuit neck torsion test, more research is required into the methodology and specifically the method of analysis. In general, the SPNT test has the best potential for differential diagnosis compared to eye head coordination.

## Conclusion

In the majority of studies included in this review, patients show altered eye reflexes and smooth pursuit movements which may impair the coordination of head and eyes.

In this review three methods of eye movement examination are found. The used methods and the patient populations significantly differ. At present there is not one single test that provides the required information. A specific combination of tests may be more suitable to properly determine eye motion.

At the present time, the head-eye coordination tests may be the most suitable method for clinical use. Further studies of eye stabilization reflexes can help to clarify the aetiology of oculomotor problems in patients with WAD. More research into the methodology of the SPNT test is required to evaluate the clinical value.

## Abbreviations

COR: Cervico-ocular reflex
OKR: Optokinetic reflex
SHEM: Sequential head and eye movement
SIGN: Scottish Intercollegiate Guidelines Network
SPNT: Smooth pursuit neck torsion
SPNTdiff: The difference between the gain in neutral and in relatively rotated position
VOR: Vestibulo-ocular reflex
WAD: Whiplash associated disorders
